# The role of hormonal contraceptive use in mediating sociodemographic predictors of overweight/obesity among women of reproductive age in Ghana

**DOI:** 10.1371/journal.pgph.0004823

**Published:** 2025-11-04

**Authors:** Shaibu Issifu, Lydia Sarponmaa Asante, Issah Sumaila, Debora Awuah Appietuah, Anthony Twum, Helen Agodzo, Michael Opoku-Mireku

**Affiliations:** 1 School of Public Health and Allied Science, Catholic University of Ghana, Fiapre, Ghana; 2 Kintampo Municipal Hospital, Ghana Health Service, Bono East, Ghana; 3 Bono East Regional Health Directorate, Ghana Health Service, Bono East, Ghana; 4 Atebubu-Amantin Municipal Health Directorate, Ghana Health Service, Bono East, Ghana; University of Ghana, GHANA

## Abstract

The global rise in overweight and obesity poses significant public health challenges, contributing to premature mortality and preventable disabilities. Moreover, efforts to increase reproductive autonomy through the promotion of modern contraceptive methods are underway, with persisting concerns regarding their potential influence on weight gain. This study aimed to identify the risk factors associated with overweight and obesity and to examine the mediating role of hormonal contraceptive usage among Ghanaian women of reproductive age. An analytical cross-sectional study was conducted using secondary data from the 2022 Ghana Demographic and Health Survey, which involved 6,181 women aged 20 years and above. The data was cleaned and analysed with Stata Corp version 18. Associations between overweight/obesity and various sociodemographic factors were assessed, and survey-adjusted logistic regression model was used to estimate adjusted odds ratios. Survey-adjusted mediation analysis was performed via structural equation modelling to evaluate the indirect effects of hormonal contraceptives use on the relationships between the independent variables and BMI. The analysis revealed that factors such as age, highest educational attainment, marital status, religion, residential status, region, nature of employment, and wealth quintile were significantly associated with high BMI with the exception of contraceptive usage. The results of the mediation showed that, hormonal contraceptives had no significant indirect role on the relationship between high BMI and its covariates. The study revealed several determinants of high BMI, however, hormonal contraceptives neither directly nor indirectly influenced high BMI. Whereas, there is the need to integrate weight management counselling into family planning for general well-being, there is the need to intensify education to dispel misconception about the weight gain associated with the usage of hormonal contraceptives to maximize the uptake of contraceptives and improve the overall health of women in Ghana.

## Background

Obesity is characterized by excessive fat accumulation that impairs health [1]. The prevalence of obesity is a significant global health concern because of its association with the pathogenesis of various noncommunicable diseases (NCDs), such as cardiovascular diseases, type 2 diabetes, and certain cancers [2]. Additionally, obesity is the fifth (5^th^) leading risk factor for disease burden globally [3]. The body mass index (BMI), also known as the Quetelet index, is commonly used as a simple and cost-effective measure for classifying overweight and obesity in adults [4,5]. According to the World Health Organization, in 2021, high BMI was responsible for approximately 4 million deaths from NCDs worldwide [6].

The prevalence of obesity has been increasing globally, with more than a billion people classified as obese as of 2022 [6]. This increase is attributed to factors such as the widespread availability of energy-dense [7] foods, nutrient-poor foods [8], sedentary lifestyles, and urbanization [9]. In particular, women are disproportionately affected [10], partly due to biological factors [11], sociocultural influences [12], inappropriate dietary habits [7] and physical inactivity [13].

The burden of high BMI in Ghana has been increasing at an alarming rate among women with potentially dangerous consequences with regards to noncommunicable disease [14,15]. The prevalence of overweight increased from 29.9% in 2008 [16] to 50.2% in 2022 [17]. The Ghana Steps Report also revealed a higher BMI rate of 1 in every 2 persons aged 18–69 years. The rising prevalence of overweight corresponds with that of non-communicable diseases, for instance the national prevalence of hypertension among women was 12.9% in 2014 [18] but rose to 25.3% in 2022 [17]. The rising burden of high BMI in Ghana has been partly attributed to urbanization [19], unfavorable food environment [8], increasing wealth quintile [17], and marital status among others [14,20]

Concurrently, efforts to promote modern contraceptive use have been intensified to enhance good reproductive health outcomes [21]. Modern contraceptives empower women to make informed decisions about childbearing, thereby preventing unsafe abortion, reducing maternal mortality and improving child health [22]. However, in sub-Saharan Africa, including Ghana, the use of modern contraceptives remains low and is hindered by misconceptions, cultural beliefs, and limited access to healthcare services [23]. Whereas the global uptake is 77% among the target population, sub-Saharan Africa is still lagging, with 56% coverage [21]

One prevalent perception is that hormonal contraceptives lead to significant weight gain, which deters women from their use [24,25]. While some studies suggest a potential association between hormonal contraceptive use and weight changes [26], the evidence is inconclusive [27,28]. Weight changes are often modest [27,29], with some studies outrightly rejecting a causal association between oral contraceptives and weight gain [30,31].

Given the increasing obesity rates [6] and the need to also improve contraceptive uptake, it is imperative to explore the relationship between BMI and hormonal contraceptive use [24]. Understanding this relationship can inform public health strategies aimed at addressing obesity while promoting effective contraceptive usage. Therefore, this study seeks to examine the determinants of higher BMI and assess the potential mediating role of hormonal contraceptive use among women of reproductive age in Ghana.

## Methodology

### Study design

This study analyses secondary data from the nationally representative 2022 Ghana Demographic and Health Survey (GDHS) conducted by the Ghana Statistical Service and partners. The analysis focused on women aged 20–49 years to examine health and demographic indicators across Ghana’s 16 administrative regions.

### Study population

The study population comprised Ghanaian women aged 20 years and above who participated in the 2022 GDHS. This nationally representative survey was conducted by the Ghana Statistical Service (GSS) between October 17, 2022, and January 14, 2023, with technical assistance from the ICF through the DHS Program. Whilst the GDHS primarily targets women aged 15–49 years, this study focused on women aged 20 years and above and used the available dataset [32,33].

### Inclusion and exclusion criteria

Women aged 20–49 years who had their weight and height figures measured were included in the analysis, whereas, those below 20 years with missing, “not present,” “refused,” or “other” weight and height figures were excluded.

### Study variables

#### Dependent variables.

**Body mass index (BMI)** – Body mass index was computed from weight and height as the ratio of weight in kilogram to the square of height in meters and classified into two categories: normal (<25 kg/m²) and overweight/obese (high BMI) (≥25 kg/m²).

#### Mediating variable.

**Contraceptive usage** – The contraceptive status was dichotomized based on whether the respondent was using a hormonal contraceptive or not (non-hormonal/no contraceptive). Hormonal contraceptives include all family planning commodities containing any synthetic hormone (estrogen or progesterone), such as pills, injectables, implants, emergency contraceptive pills, and other modern methods as defined in the Demographic and Health Survey (DHS) program data. Respondents who were using non-hormonal methods or not using any family planning method at all were categorized as non-hormonal/no contraceptive.

### Independent variables

#### Sociodemographic characteristics.

Age was categorized into two (2) groups: ideal childbearing age (20–35 years) and above ideal childbearing age (36–49 years). The 16 administrative regions of Ghana were consolidated into three belts: northern (Northern, Savanna, Upper East, Upper West, and Northeast), middle (Bono, Bono East, Ahafo, Ashanti, and Eastern), and southern (Western, Western North, Central, Greater Accra, Oti, and Volta). Other variables, including religion, were also recategorized into four (Christian, Islamic, traditional, and atheist), residential status was dichotomized into rural and urban. Other variables are marital status categorized into never in union (those who had never been in any relationship), married, cohabitating, not in relationship (those who were previously in relation but have divorced, or their partners have passed away); wealth quintile with the five categories; nature of employment, categorized into three as follow: for family members, for someone else, and self-employed.

### Sampling technique

The dataset utilized for this study was the **GHIR8CFL** file from the 2022 GDHS. This dataset comprised information from 15,014 women aged 15–49 years.

To ensure the reliability and generalizability of the findings, the dataset underwent meticulous cleaning via a listwise deletion approach, resulting in a final analytical sample of 6,181 respondents. The cleaning process involved the following steps:

1. Exclusion of Respondents Without Weight or Height Data: A total of 7,338 respondents lacking either weight or height measurements were excluded, as body mass index (BMI) could not be calculated for these individuals.
2. Removal of Respondents Aged Below 20 Years: Given that raw BMI is not an appropriate measure for individuals under 20 years where BMI-for-age is more suitable [17,34], 1,436 respondents in this age group were excluded.
3. Exclusion of Respondents with Invalid Weights or Height Entries: Fifty-nine (59) respondents with weight or height entries marked as “not present,” “refused,” or “other” were also excluded.

**Fig 1:**
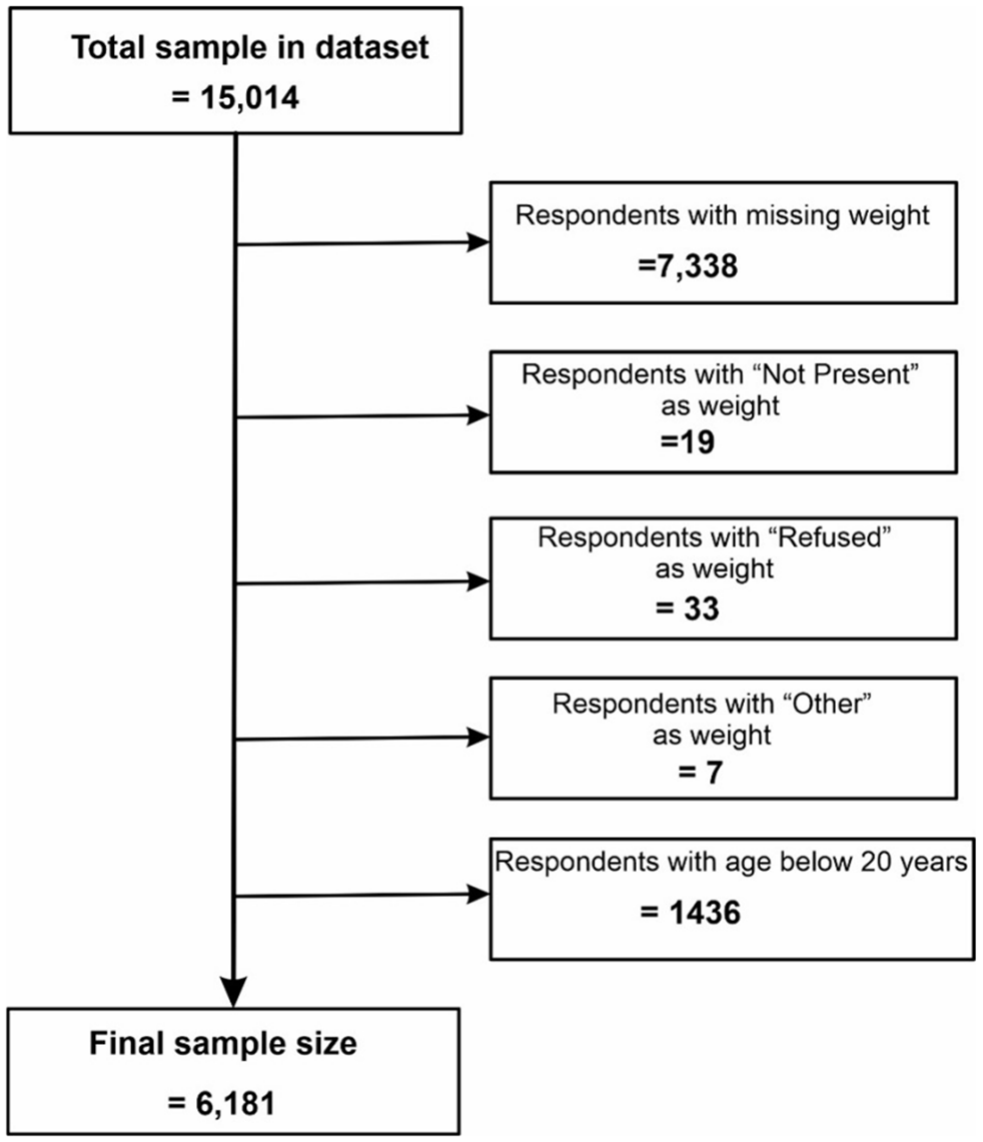
Flow chart of the data cleaning process.

Details are as shown in Fig 1.

### Sample size

Following a rigorous data cleaning process, the final analytical sample comprised 6,181 respondents, representing 41.2% of the original dataset. This indicates that 8,833 observations (58.8%) were excluded because of missing or invalid data, as detailed in the data cleaning procedures.

### Data analysis

The various independent variables used for the study were summarized and presented in a table with their p values determined via the chi-square test with row percentages. The prevalence of high BMI, along with 95% confidence intervals, was estimated via the logit proportion function. Nine (9) independent variables were examined for this study. To prevent over or under fitting of the model, lasso regression was employed to select the most appropriate predictors which selected all the 9 predictors.

To prevent inappropriate estimates and also account for the design effect, survey-adjusted multivariate logistic regression analysis was done to compute adjusted odds ratios (aORs) for each of the 9 selected predictors to evaluate their strength of association with high body mass index. The independent variables were assessed for collinearity via the variable inflation factor (VIF). All the independent variables had VIF values of less than 3 and a mean VIF of 1.32, indicating the absence of collinearity among them.

To investigate the mediating role of hormonal contraceptives in the relationship between BMI and the independent variables, a mediation analysis was conducted. Survey-adjusted structural equation modelling (SEM) was employed to construct a directed cyclic graph (DAG) representing the hypothesized mediation pathways. The analysis utilized a nonparametric robust standard error tools such as linearized variance-covariance estimator (vce) and degree of freedom (586). The indirect effects, representing the average causal mediation effects (ACME), were estimated through nonlinear combination analysis. Statistical significance was set at p < 0.05 for all analyses.

### Ethical issues

Formal authorization was obtained from the Demographic and Health Survey Program for the utilization of the dataset in this study. The GSS obtained clearance from the Ethical Review Committee (ERC) of the Ghana Health Service to ensure that the survey procedures were in accordance with Ghana’s ethical research standards. Also, ICF obtained clearance from the ICF Institutional Review Board (IRB) in accordance with U.S. and international ethical research standards.

## Results

### Sociodemographic characteristics of the respondents

The bivariate analysis in Table 1 below revealed significant associations between all the sociodemographic characteristics and body mass index (BMI) among the study participants (p < 0.001) with the exception of contraceptives. Age was positively associated with high BMI, as approximately 53.5% of individuals above 35 years of age had a high BMI, whereas 38.0% of those aged 20–35 years had a high BMI. Educational attainment was also linked to BMI; 55.7% of respondents with higher education levels had high BMIs, which was double the 27.9% observed in individuals without formal education. Marital status was another significant factor; individuals previously in a union but currently in no relationship had the highest prevalence of high BMI at 55.9%, whereas those never in a union had the lowest at 35.4%.

Christians had the highest prevalence of high BMI (46.9%), followed by Muslims (39.1%) and traditionalists (15.0%). Compared with rural residency, urban residency was associated with a higher BMI (32.8% vs 54.9%). Regionally, the middle and southern belts reported higher proportions of individuals with high BMIs (51.2% and 51.9%, respectively) than did the northern belt (28.5%).

In terms of the nature of employment, self-employed individuals presented a higher prevalence of high BMI, at 49.2%. Additionally, there was a consistent increase in the prevalence of high BMI across ascending wealth quintiles, ranging from 18.6% in the poorest group to 68.0% in the richest group.

**Table 1:** Association between body mass index and the sociodemographic characteristics.

Variable	Normal	Overweight/obesity	Total	p value
N	3475 (%)	2698 (%)		
**Age (years)**				<0.001
20 - 35	2,418 (62.0)	1,485 (38.0)	3903	
36 -49	1,060 (46.5)	1,218 (53.5)	2278	
**Highest educational level**				<0.001
No education	1155 (72.1)	445 (27.9)	1602	
Primary	482 (54.1)	409 (45.9)	891	
Secondary	1558 (51.1)	1491 (48.9)	3049	
Higher	283 (44.3)	356 (55.7)	639	
**Marital status**				<0.001
Never in union	753 (64.6)	413 (35.4)	1166	
Married	1928 (56.1)	1509 (43.9)	3437	
Cohabitating	522 (54.7)	432 (45.3)	954	
No longer in relationship	275 (44.1)	349 (55.9)	624	
**Religion**				<0.001
Traditional	108 (85.0)	19 (15.0)	127	
Christian	2277 (53.1)	2009 (46.9)	4286	
Islam	995 (60.9)	639 (39.1)	1634	
Atheist	98 (73.1)	36 (26.9)	134	
**Type of place of residence**				<0.001
Rural	2102 (67.2)	1028 (32.8)	3130	
Urban	1376 (45.1)	1675 (54.9)	3051	
**Region**				<0.001
Northern belt	1500 (71.5)	597 (28.5)	2097	
Middle belt	952 (48.8)	997 (51.2)	1949	
Southern belt	1026 (48.1)	1109 (51.9)	2135	
**Nature of employment**				<0.001
For family member	500 (73.0)	185 (27.0)	685	
For someone else	652 (55.0)	534 (45.0)	1186	
Self-employed	1740 (50.8)	1683 (49.2)	3421	
**Contraceptives**				0.325
Non-hormonal/no contraceptive	2,780 (56.58)	2,133 (43.42)	4,913	
Hormonal	698 (55.05)	570 (44.95)	1,268	
**Wealth index combined**				<0.001
Poorest	1200 (81.4)	274 (18.6)	1474	
Poorer	860 (64.6)	471 (35.4)	1331	
Middle	624 (51.6)	586 (48.4)	1210	
Richer	477 (40.6)	698 (59.4)	1175	
Richest	317 (32.0)	674 (68.0)	991	

### The determinants of body mass index (BMI)

Table 2 below shows the fitting of adjusted odds ratios to measure the strength of the associations between body mass index and the independent variables. All the variables showed various degrees of association with BMI with the exception of contraceptives. Holding all other variables constant, the odds of overweight/obesity were approximately 70% greater (aOR: 1.68, 95% CI: 1.42, 2.00, p < 0.001) for respondents 36–49 years than for those younger than 36 years. After adjusting for confounders, compared with those who had no education, the odds of having a high BMI among those who had various educational levels were as follows: primary education (aOR: 1.64, 95% CI: 1.21, 2.21, p = 0.001); secondary education (aOR: 1.52, 95% CI: 1.18, 1.96, p = 0.001); and higher education (aOR: 1.82, 95% CI: 1.30, 2.55, p < 0.001). Compared with those of respondents who had never been in any union, the odds of having a high BMI were approximately 65% and 66% greater for respondents who were married (aOR: 1.65, 95% CI: 1.31, 2.08, p < 0.001) and cohabitating (aOR: 1.66, 95% CI: 1.28, 2.16, p < 0.001), respectively. The odds were, however, 2 times greater among respondents who were no longer in any relationship (aOR: 2.12; 95% CI: 1.56, 2.88; p < 0.001). In terms of religion, whereas Christians had 2.6 times greater odds of being overweight/obese (aOR: 2.63, 95% CI: 1.27, 5.46; p = 0.010), Muslims had 3.3 times (aOR: 3.29, 95% CI: 1.58, 6.84; p = 0.001) greater odds than traditionalists did. Being in an urban residence was associated with a higher BMI, with approximately 36% greater odds than being in a rural residence (aOR: 1.36, 95% CI: 1.13, 1.64; p = 0.001). Additionally, respondents in the middle (aOR: 1.94, 95% CI: 1.57, 2.40, p < 0.001) and southern belts (aOR: 1.71, 95% CI: 1.36, 2.14, p < 0.001) of Ghana had 94% and 71% higher odds of overweight/obesity, respectively, than did respondents in the northern belt. Holding all other factors constant, the odds of overweight/obesity were 63% greater among respondents who were self-employed than among those who were working for family members (aOR: 1.63, 95% CI: 1.27, 2.10, p < 0.001); however, there was no evidence of a significant difference between respondents who worked for other people and those who worked for their family members. There were no significant differences respondents using hormonal or non-hormonal/no contraceptives. When adjusted for confounders, the odds of overweight/obesity increased with increasing wealth compared with the poorest as follows: poorer (aOR: 1.73, 95% CI: 1.32, 2.28, p < 0.001); middle (aOR: 2.36, 95% CI: 1.72, 3.39, p < 0.001), richer (aOR: 4.06, 95% CI: 2.89, 5.71, p < 0.001), and richest (aOR: 5.42, 95% CI: 3.72, 7.88, p < 0.001).

**Table 2:** Survey-adjusted multivariate logistic regression analyses of body mass index (BMI) and its covariates.

Variable	aOR	p-value	95%CI
**Age**			
20–35years	1		
36 years & above	1.684	<0.001	1.415, 2.004
**Highest education level**			
No education	1		
primary	1.635	0.001	1.213, 2.205
secondary	1.522	0.001	1.179, 1.963
higher	1.823	<0.001	1.302, 2.551
**Marital status**			
Never in union	1		
Married	1.65	<0.001	1.309, 2.08
Cohabiting	1.663	<0.001	1.279, 2.163
Not in relationship	2.117	<0.001	1.558, 2.877
**Religion**			
Traditional	1		
Christian	2.628	0.01	1.265, 5.459
Islam	3.29	0.001	1.584, 6.835
Atheist	1.469	0.403	0.595, 3.624
**Region**			
Northen Belt	1		
Middle Belt	1.94	<0.001	1.569, 2.398
Southern Belt	1.705	<0.001	1.359, 2.139
**Type of place of residence**			
Rural	1		
Urban	1.36	0.001	1.126, 1.643
**Nature of employment**			
For family member	1	.	
for someone else	0.893	0.47	0.656, 1.215
self-employed	1.632	<0.001	1.266, 2.104
**Contraceptive**			
Non-hormonal/no contraceptive	1	.	
Hormonal	1.199	0.078	0.980, 1.468
**Wealth index combined**			
poorest	1	.	
poorer	1.733	<0.001	1.317, 2.28
middle	2.361	<0.001	1.715, 3.251
richer	4.058	<0.001	2.885, 5.708
richest	5.416	<0.001	3.723, 7.879

### The mediating role of hormonal contraceptives in the relationship between body mass index and its covariates

**Fig 2:**
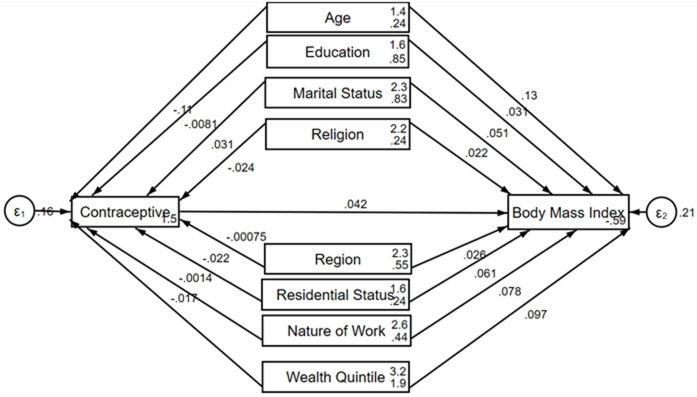
Directed acyclic graph (DAG), showing the indirect relationship between body mass index and its covariate through contraceptives.

The mediation analysis shown in Fig 2, depicted in the directed acyclic graph (DAG), explored the relationships between body mass index (BMI) and various sociodemographic factors, with hormonal contraceptive use serving as a potential mediator. There was non-significant direct association between hormonal contraceptive use and BMI (b = -0.042, p = 0.056). Age was positively associated with BMI; individuals aged 36–49 years were more likely to have a higher BMI than those aged 20–35 years were (c_1_ = 0.13, p < 0.001). However, age was negatively associated with hormonal contraceptive use, indicating that older individuals were less likely to use hormonal contraceptives (a_1_ = -0.108, p < 0.001). Marital status also showed a direct positive association with BMI, with individuals in any form of union being more likely to have a higher BMI than those never in a relationship (c_3_ = 0.05, p < 0.001). Additionally, marital status was positively associated with contraceptive use (a_3_ = 0.031, p < 0.001). Notably, there was a significant direct association between all the independent variables and body mass index with the exception of contraceptive and religion. In addition to age and marital status, wealth quintile was significantly associated with contraceptive when adjusted for BMI (a_8_ = -0.017, p = 0.033).

The results of the non-linear combination analysis presented in Table 3 shows that hormonal contraceptive use does not significantly influence indirectly the relationship between BMI and its independent variables.

**Table 3:** Indirect effect of contraceptive use on the relationship between BMI and its factors.

Variable	Coefficient	P value	[95% conf. Interval]
Age	-0.00451	0.058	-0.00918, 0.00016
Highest education level	-0.00034	0.448	-0.00121, 0.00054
Marital status	0.00130	0.089	-0.00020, 0.00279
Religion	-0.00101	0.262	-0.00276, 0.00075
Region	-0.00003	0.941	-0.00087, 0.00081
Residence status	-0.00093	0.346	-0.00287, 0.00101
Nature of work	-0.00006	0.900	-0.00098, 0.00086
Wealth Quintile	-0.00070	0.169	-0.00169, 0.00030

## Discussion

This study revealed a significant direct association between higher body mass index (BMI) and all the independent variables with the exception of contraceptives. To the best of our knowledge, this is the first study that seeks to explore the indirect effect of hormonal contraceptive use on the relationship between BMI and its independent variables in Ghana. The results of the study shows that contraceptive use is neither a significant predictor of high BMI nor has an indirect influence in the relationship between BMI and its covariates. This implies that hormonal contraceptives play no role in one becoming overweight/obese.

The prevalence of overweight/obesity among the respondents was 43.7% (95% CI: 42.5%, 45.0%), which is consistent with the global prevalence of 43% reported by the WHO [6]. In Ghana, this result confirms finding of studies by the 2022 Ghana demographic and health survey which reported 50.2% [17], Tette et al. [35] and Lartey et al. [15]. The prevalence was, however, slightly higher than the findings of the Ghana Steps Report on the noncommunicable disease risk factor assessment which reported a prevalence of 34.3% [36], Obirikorang et al. [37] reported 31% among undergraduate students, and a systematic review and meta-analysis by Yussif et al. [12] revealed an overweight prevalence of 23.1%. The differences in the results of this study and those of Obirikorang et al. and Yussif et al. could be attributed to the use of a youthful population and the research design respectively. Compared with their younger counterparts, women aged above 35 years presented 68% greater odds of having high BMI, underscoring the influence of age on BMI. This finding aligns with previous studies conducted in Ghana [38], the United States [39], Pakistan [40], and Spain [41], all of which reported a positive association between increasing age and higher BMI. The observed trend may be attributed to the reduced physical activity associated with aging due to increased responsibilities [42] and economic factors [20].

This study revealed a positive association between education and high BMI; the higher the level of education was, the greater the odds of high BMI. The finding of educational status confirms that of John Tetteh et al. in Ghana [35], whose study reported approximately 2 times greater odds among respondents who had completed tertiary education than among those who had not. Additionally, Jimenez-Mora et al. [10] reported an increasing prevalence of overweight with higher education in Colombia. In Iran, Zare-Zardiny et al. [43] reported a positive correlation between educational status and BMI. The likely reason for the higher odds of high BMI for respondents with higher education is their employability as reported in the Ghana living standard survey 7, especially those who had completed tertiary education, which equipped them financially with the ability to buy what they want including highly processed foods [44].

Participants currently married were 65% more likely to have a high BMI than those who had never been in a union. This observation is consistent with findings of Nikolic Turnic et al. [45], who reported high BMI among married individuals. Similarly, Amegah et al. [38], in a hospital-based study in Cape Coast, Ghana, noted higher odds of having a high BMI among married respondents. Potential explanations include social support from spouses [20], a sense of security [45], reduced period of physical activities due to matrimonial responsibilities [42] and cultural perceptions valuing larger body sizes within marriages [12].

Urban residents demonstrated increased odds of having an high BMI, corroborating global findings [9] and studies from Ghana [14,19,35,36] and Nigeria [46]. The higher odds in urban areas could be as a result of the high employment rate in urban areas, which equips them financially to purchase all that they want, including high-caloric foods [44]. This trend may also be linked to increased availability and consumption of ultra-processed foods in urban areas and a high propensity for a sedentary lifestyle [7–9]. Furthermore, the traditional occupation of rural dwellers is agriculture, which is physically intensive, therefore providing them with the necessary amount of physical activity [9]. To reduce the prevalence of obesity among urban dwellers, there is a need to promote a good built environment that promotes physical activities [47] and also encourage workplace-based physical activities [48]. Additionally, respondents from the middle and southern belts presented 94% and 71% higher odds of having high BMI compared to those from the northern belt, respectively. Economic and environmental disparities between these regions may limit access to resources, which tends to be a protective factor for high BMI [49]. For example, the Ghana Standards Living Survey 7 [44] report revealed a very low employment rate in the northern belt (15.5%) compared with the middle belt (26.6%) and southern belt (29.5%), which implies low purchasing power, thereby limiting their ability to purchase and consume highly processed foods.

The wealth quintile also showed a significant association with higher BMI, with odds increasing alongside wealth status, peaking among the richest individuals. This finding aligns with Dogbe [50], whose study utilized data from the Ghana Living Standards Survey 7. Similar associations have been reported in India [51] and Ethiopia [52]. A plausible explanation is that individuals in higher wealth quintiles have greater purchasing power, potentially leading to increased consumption of calorie-dense foods [53]. This underscores the impact of economic disparities on the prevalence of overweight/obesity.

Importantly, this study revealed that hormonal contraceptives neither had direct nor indirect influence on body mass index of Ghanaian women of reproductive age. This finding agrees with that of Procter-Gray et al. [27] whose study reported no association between intake of oral contraceptives and weight gain, however, that study used young female runners. Furthermore, Gallo et al. [30] reported no significant associations between oral contraceptive use and weight gain. One plausible explanation is that estrogen has been reported to increase resting energy expenditure by up to 208 kcal, potentially offering a protective effect against high BMI [54]. On the contrary, this study disagrees with that of Avenant et al. which established strong associations between hormonal contraceptives and weight gain [28], the study reported a 5% weight increase over baseline after 12 months of etonogestrel implant use, particularly among women under 40 years of age. Also, a longitudinal study reported an average weight gain of 3.8 kg after 24 months of implant use [55]. This study therefore implies that hormonal contraceptive usage does not significantly influence weight gain contrary to the fears expressed by a section of the population [26]. This finding highlights the importance of strengthening targeted education on the safety of contraceptives to dispel the misconceptions, help boost uptake and enhance the reproductive autonomy among the Ghanaian women.

### Limitations

This study is limited by BMI’s inability to assess fat distribution or distinguish fat from muscle. Self-reported data may introduce bias, and the lack of waist and hip measures hinders fat distribution analysis. Lastly, the cross-sectional design nature of the data makes it impossible to make causal inferences between variables. Furthermore, not all confounders of BMI were adjusted for including physical activity level, dietary lifestyle or habit, obstetric factors, and pre-existing medical conditions that have impact of body weight.

### Strength of the study

The strength of the paper is the higher sample size which gives the needed statistical power and the precision required. Also, the statistical analysis used factor in the design effect of the survey preventing over or under estimation of type I error.

## Conclusion

This study underscores the multifaceted determinants of high body mass index (BMI) among Ghanaian women of reproductive age and its interplay with hormonal contraceptive use. The key predisposing factors of overweight/obesity include older age (36–49 years), higher education level, being married, cohabiting, or previously in a relationship, being a Christian or a Muslim, urban residence, higher wealth quintiles, and regional disparities (middle and southern belts). However, contraceptive was neither a determinant of high BMI nor indirectly influenced the relationship between BMI and its covariates. The findings suggest that public health interventions aimed at addressing high BMI among Ghanaian women has to take into consideration the complex determinants. Whereas, healthy diets and lifestyle modifications aimed at addressing weight gain in women is generally advisable, behavioural change communication aimed at highlighting the benefits of contraceptives and dispelling some misconceptions associated with contraceptives is needed. This strategy can promote contraceptive uptake, enhance women’s reproductive autonomy, and improve overall health outcomes among Ghanaian women.

Future research should explore longitudinal study on the influence of hormonal contraceptives on high BMI and assess its long-term impacts on health outcomes of women in Ghana.

## Supporting information

S1 Data The dataset that was used for the study. (DTA)

S1 File Strobes checklist. (DOCX)
